# Maternal and fetal cardiovascular and metabolic effects of intra-operative uterine handling under general anesthesia during pregnancy in sheep

**DOI:** 10.1038/s41598-020-67714-y

**Published:** 2020-07-02

**Authors:** Caroline J. Shaw, Kimberley J. Botting, Youguo Niu, Christoph C. Lees, Dino A. Giussani

**Affiliations:** 1 Department of Physiology, Development and Neuroscience, University of Cambridge, Cambridge, CB2 3EG UK; 2 Institute of Reproductive and Developmental Biology, Imperial College London, London, UK; 3 Cambridge Cardiovascular Research Initiative, Cambridge, UK; 4 Department of Obstetrics and Gynecology, University Hospitals Leuven, Leuven, Belgium

**Keywords:** Physiology, Medical research

## Abstract

A cohort study of 6,500,000 human pregnancies showed an increased risk of adverse fetal outcomes following abdominal but not non-abdominal surgery under general anesthesia. This may be the consequence of uterine handling during abdominal surgery. However, there are no data on any effects on the cardiometabolic physiology of the fetus or mother in response to uterine manipulation in otherwise healthy pregnancy. Consequently, 9 sheep in late gestation were anesthetized with isofluorane and maternal and fetal catheters and flow probes were implanted to determine cardiovascular and metabolic changes during uterine handling. Uterine handling led to an acute increase in uterine artery vascular resistance, fetal peripheral vasoconstriction, a reduction in oxygen delivery to the femoral circulation, worsening fetal acidosis. There was no evidence of systemic fetal hypoxia, or changes in fetal heart rate, carotid blood flow or carotid oxygen delivery. Therefore, the data support that uterine handling during abdominal surgery under general anesthesia can impact adversely on fetal cardiometabolic health. This may provide a potential explanation linking adverse fetal outcomes in abdominal compared with non-abdominal surgery during pregnancy. The data have important implications for human fetal surgery where the uterus is handled, as operative procedures during late gestation under general maternal anesthesia become more prevalent.

## Introduction

A recent retrospective cohort study of 6.5 million pregnancies in the UK found that 0.7% women underwent surgery during pregnancy for a non-obstetric indication between 2002 and 2012; of these 26% underwent abdominal surgery under general anesthesia^1^. The same study also demonstrated that there is an increased risk of miscarriage and stillbirth following abdominal surgery during pregnancy that is not found in women undergoing orthopedic, breast, ENT or perianal surgery^1^. A 2005 systematic review of 54 studies involving 12,452 pregnant women also found an increased risk of surgery-induced labor, and that the risk of fetal loss was significantly raised when abdominal surgery was complicated by peritonitis^2^. These data suggest the hypothesis that there is a potential adverse effect on the fetus of abdominal surgery, which predisposes to pregnancy loss, above and beyond the effects of prolonged general anesthesia and/or maternal systemic illness or injury.

Although laparoscopy is increasingly used during pregnancy, the size of the gravid uterus in the second and third trimester means laparotomy remains more common^3^, and open abdominal surgery was performed in 66% of second and third trimester operations in the UK retrospective cohort study^1^. Regardless of abdominal approach, the surgical requirements to access the maternal intra-abdominal structures mean there is an inevitable degree of uterine handling or pressure.

However, for sound ethical reasons, there are currently no data available in humans regarding any effects on the cardiovascular or metabolic physiology of the fetus and of the mother in response to uterine handling in otherwise healthy pregnancy. Therefore, in this study, we have used the well-established surgically instrumented pregnant sheep model with catheters and flow probes^4–7^ to determine the effects of uterine handling on the cardiovascular and metabolic status of a healthy mother and fetus in late gestation, under general anesthesia.

## Materials and methods

All procedures were performed under the UK Animals (Scientific Procedures) Act 1986 and were approved by the Ethical Review Board of the University of Cambridge.

### Surgical preparation

Nine pregnant Welsh mountain sheep carrying singleton fetuses at 116 ± 1 days of gestation (term ~ 147 days) were used. Food but not water was withheld from the pregnant ewe for 24 h prior to operation and surgery was performed under aseptic conditions using well-established techniques^4–7^. Anesthesia was induced with alfaxalone 3 mg kg^−1^ I.V. (Alfaxan, Jurox); no pre-medication was administered. The ewe was intubated with a size 7.0–9.0 cuffed endotracheal tube dependent on maternal weight under the guidance of an illuminated laryngoscope. General anesthesia was maintained with isoflurane (1.5–2.5% in 4:1 O_2_:N_2_O) administered by mechanical ventilation (settings: positive pressure, respiratory rate 16–20, tidal volume 6–8 ml kg^−1^, typically 250–350 ml) throughout the operation. Non-invasive maternal monitoring of maternal oxygen saturation and end-tidal carbon dioxide (EtCO_2)_ was performed throughout the period of anesthesia and SpO_2_ was maintained > 94% and EtCO_2_ was maintained < 6%. Minute volume settings were adjusted as required to maintain the ewe within these physiological limits; there was no attempt to super-oxygenate the ewes. Carprofen (1 mg kg^−1^, Rimadyl, Pfizer), a veterinary non-steroid anti-inflammatory drug, was given at 50% total anesthetic time to aid maintenance of anesthesia; this drug is not known to affect materno-fetal cardiovascular status^7^. The ewe was positioned in dorsal recumbency and a left lateral tilt on the operating table of approximately 30° was maintained to reduce autocaval compression by the gravid uterus; maternal mean arterial blood pressure was maintained > 60 mmHg with a crystalloid infusion (0.9% NaCl, Aquapharm, Animalcare) at a rate of 5 ml kg^−1^ h^−1^. Following abdominal midline and uterine incisions, fetal arterial catheters were introduced into the ascending and descending aorta via the fetal carotid and femoral arteries, respectively. A third catheter was secured to the fetal hind limb for monitoring of intrauterine amniotic pressure. Time-transit flow probes were placed around the contralateral fetal carotid and femoral arteries (2 mm aperture, R-series, Transonic Systems Inc., Ithaca, NY) and on the maternal uterine artery at the level of the cervix (4 mm aperture, S-series, Transonic Systems Inc., Ithaca, NY, USA). An arterial catheter was also introduced into the maternal descending aorta via the femoral artery. Catheters were filled with heparinized saline (100 IU ml^−1^ heparin sodium in 0.9% NaCl solution) and connected to pressure transducers (ArgoTrans, Argon Medical Devices Inc.). These surgical procedures in the mother and fetus are well established and routine in our laboratory, and if the mother and fetus were allowed to recover from anesthesia, both would achieve complete post-surgical recovery within 5 days^4–6, 8^. In the present study, since recording of cardiovascular function was necessary during general anesthesia before, during and after uterine manipulation, the abdominal incision was left open, and catheters and flow probes were connected intra-operatively to a customized data acquisition system, the Cambridge Data Acquisition System (CamDAS)^5^. Using this system, the absolute values for pressure and blood flow signals in the mother and fetus could be converted to digital signals and sampled at a rate of 500 kHz (IDEEQ, Maastricht Instruments). Heart rate was derived from the arterial pressure pulsatility. The data were recorded for offline analysis.

### Experimental protocol

Immediately after surgical instrumentation, during general anesthesia, the experimental protocol was divided into a baseline period of 30 min during which the uterus was not touched, followed by 30 min of uterine manipulation. This comprised gentle manual handling of the body of the uterus without rotation and light pressure, comparable to packing or retraction. There was then a further 30-min recovery period (Figure S1). During the 90-min experimental protocol, there was continuous recording of maternal and fetal arterial blood pressure and heart rate, uterine artery blood flow and fetal femoral and fetal carotid artery blood flow. In addition, blood samples (0.3 ml) were taken from the maternal femoral artery and the fetal femoral and carotid arterial catheters at the following time points: (1) the start of experimental protocol (time point “− 30”); (2) the start of uterine manipulation (time point “0”); (3) after 15 and 30 min of uterine manipulation (time points “15”, “30”); and (4) at the end of the recovery phase (time point “60”). These blood samples were used to determine acid–base status and partial pressures of oxygen and carbon dioxide (ABL5 Blood Gas Analyzer, Radiometer) as well as hemoglobin, hematocrit and oxygen saturation of the blood (ABL80 Flex, Radiometer; blood samples corrected for maternal and fetal temperature). Arterial lactate concentrations were obtained from an automated analyzer (YSI 2300 Stat Plus, Yellow Springs Instruments). At the end of the experimental protocol ewes were euthanized by terminal anesthesia.

### Data and statistical analyses

Only 1 experiment was conducted in any one preparation. Each animal served as its own control with cardiovascular recording before, during and after surgical manipulation. All experiments were performed at the same time of the day and during the same experimental season. Changes in vascular resistance were calculated adopting Darcy and Ohm’s principles governing hemodynamics by dividing arterial blood pressure by blood flow in the respective circulation^4, 5^. Recordings of continuous cardiovascular data for each variable were converted into mean values for each sequential minute (“minute means”) of the experimental protocol (Labchart 7 Pro, Ad Instruments Ltd.). Minute means (+ S.E.M) of cardiovascular data were then expressed as absolute or as percent change from baseline. A summary measure of the analysis (area within the curve for each time period) was then applied to the serial cardiovascular data to focus statistical comparisons between time periods^9^. A repeated measures (RM) one-way ANOVA was then used to assess the effect of different time periods within the experimental protocol on cardiovascular and metabolic data. Statistical significance for comparisons was accepted when *P* < 0.05 using a Greenhouse–Geisser correction. Significant differences were isolated using the post-hoc Bonferroni test.

## Results

### Maternal cardiovascular responses

**Figure 1:**
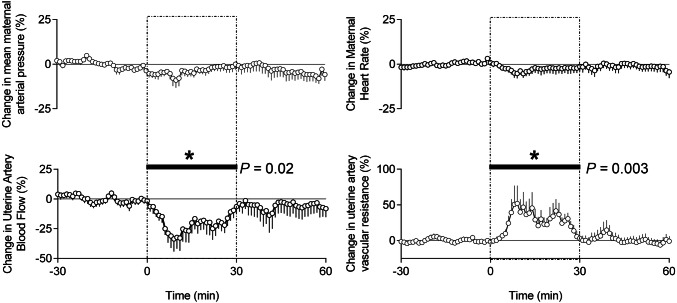
Maternal cardiovascular responses to uterine manipulation. The graph shows mean values for each sequential minute ± SEM (n = 9) of percentage change from baseline (− 30 to 0 min) period. The dashed box (0–30 min) denotes the period of uterine handling. The recovery period is 30–60 min. The black bar shows the timing of significant change from baseline, assessed using a repeated measure one-way ANOVA with post hoc Bonferroni test. The *P* value shown on the graph is the overall significance of the RM ANOVA with Glasshouse–Geisser correction for the effect of time.

Basal recording commenced during steady conditions 162 ± 10 min after the onset of maternal exposure to isoflurane. During the basal period of the experimental protocol, the maternal mean arterial blood pressure was 72.8 ± 0.3 mmHg, mean maternal heart rate was 96.3 ± 0.2 bpm, mean uterine blood flow was 276 ± 1.5 ml min^−1^ and mean uterine artery vascular resistance was 0.33 ± 0.002 mmHg (ml min^−1^)^−1^. Values for maternal arterial blood pressure, maternal heart rate, uterine blood flow and uterine vascular resistance during baseline recording were appropriate for this breed of sheep under prolonged general isofluorane anesthesia (Fig. 1). During uterine handling, while maternal arterial blood pressure and heart rate were unaltered from baseline, there was an acute fall in uterine artery blood flow, secondary to an acute increase in uterine arterial vascular resistance. The significant decrease in uterine blood flow reached a nadir of − 34% below baseline 9 min after uterine handling commenced. This was associated with a peak increment in uterine artery vascular resistance of 52% above baseline (Fig. 1). Both maternal uterine artery blood flow and uterine vascular resistance returned towards baseline during the recovery period, after uterine handling was stopped.

### Fetal cardiovascular responses

**Figure 2:**
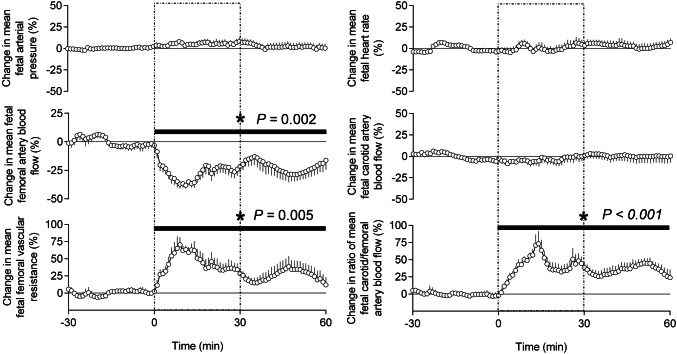
Fetal cardiovascular responses to uterine manipulation. The graph shows mean values for each sequential minute ± SEM (n = 9) of percentage change from the baseline (− 30 to 0 min) period. The dashed box (0–30 min) denotes the period of uterine handling. The recovery period is 30–60 min. The black bar shows the timing of significant change from baseline, assessed using a repeated measure one-way ANOVA with post hoc Bonferroni test. The *P* value shown on the graph is the overall significance of the RM ANOVA with Glasshouse–Geisser correction for the effect of time.

During the basal period of the experimental protocol, the fetal mean arterial blood pressure was 46.1 ± 0.1 mmHg, the fetal mean heart rate was 165 ± 1 bpm, the fetal mean carotid blood flow was 80.8 ± 0.7 ml min^−1^ and the fetal mean femoral blood flow was 29.4 ± 0.6 ml min^−1^. Mean fetal arterial blood pressure, mean fetal heart rate, mean fetal carotid blood flow and mean fetal femoral blood flow remained stable during basal recording at values appropriate for this breed of sheep under prolonged general isofluorane anesthesia (Fig. 2). During uterine handling, while mean fetal arterial blood pressure, mean fetal heart rate and mean fetal carotid blood flow were unaltered from baseline, there was a significant acute fall in mean fetal femoral blood flow, secondary to a significant acute increase in mean fetal femoral vascular resistance (Fig. 2). The significant decrease in fetal femoral arterial blood flow reached − 38% below baseline after 11 min of uterine handling. This was associated with a significant increase in femoral artery vascular resistance, reaching values of 70% above baseline. Both femoral artery blood flow and femoral vascular resistance remained significantly altered from baseline during the recovery period. Comparison of parallel changes in fetal blood flow in the carotid and femoral arteries, a ratio described as the fetal brain sparing index^5^, revealed a significant increase during uterine manipulation, an effect which persisted beyond the end of uterine manipulation and into the recovery period (Fig. 2).

### Maternal metabolic status

During the baseline period, values for maternal arterial blood gas and acid base status were stable and appropriate for this breed of sheep under prolonged general isofluorane anesthesia. The high level of basal maternal partial pressure of O_2_ (P_a_O_2_) reflects ventilation with oxygen. While maternal P_a_O_2_, oxyhemoglobin saturation, arterial base excess and concentrations of bicarbonate and lactate remained unaffected, there was a progressive rise in maternal arterial partial pressure of CO_2_ (P_a_CO_2_) and a corresponding fall in maternal arterial pH during the uterine handling and the recovery periods (Table 1). An increase in maternal hematocrit also occurred by the end of uterine handling, which remained elevated during the recovery period (Table 1).

**Table 1:** Maternal arterial acid–base and metabolic status.

Variable	Baseline		Uterine handling		Recovery	*P* value
	( −30 min)	(0 min)	(15 min)	(30 min)	(60 min)	
pH	7.40 ± 0.02	7.40 ± 0.02	7.39 ± 0.02	7.37 ± 0.02*	7.37 ± 0.02*	0.009
P_a_CO_2_ (mmHg)	51.6 ± 3.2	53.9 ± 4.2	57.4 ± 3.6*	59.3 ± 3.9*	59.0 ± 3.9*	< 0.001
P_a_O_2_ (mmHg)	202 ± 23	182 ± 20	168 ± 21	178 ± 25	174 ± 22	0.60
Oxyhemoglobin saturation (%)	102 ± 1	102 ± 1	102 ± 1	102 ± 1	102 ± 1	0.90
Arterial base excess (mmol l^−1^)	5.7 ± 1.6	6.3 ± 1.1	5.3 ± 0.9	6.0 ± 1.0	5.4 ± 1.1	0.70
Bicarbonate (mEq L^−1^)	31.7 ± 1.5	32.4 ± 1.3	32.4 ± 1.3	32.3 ± 1.2	31.4 ± 1.1	0.57
Lactate (mmol l^−1^)	0.7 ± 0.1	0.6 ± 0.1	0.6 ± 0.1	0.6 ± 0.1	0.6 ± 0.1	0.09
Hemoglobin (g d l^−1^)	8.9 ± 0.6	9.0 ± 0.5	9.1 ± 0.5	9.6 ± 0.3	9.7 ± 0.4*	0.04
Hematocrit (%)	24 ± 1	24 ± 1	25 ± 1	28 ± 1*	0.04	

Values represent mean ± SEM for n = 9 of maternal femoral arterial blood samples at the start (“− 30”) and end (“0”) of the baseline period; the midpoint (“15”) and end (“30”) of the period of uterine handling and the end of the recovery period (“60”). Significant differences are: *P < 0.05, for each timepoint when compared to timepoint “− 30”, assessed using a repeated-measures (RM) one-way ANOVA with post hoc Bonferroni test. The *P* value quoted in the final column is the overall significance of the RM ANOVA with Glasshouse–Geisser correction for the effect of time.

### Fetal metabolic status

**Figure 3:**
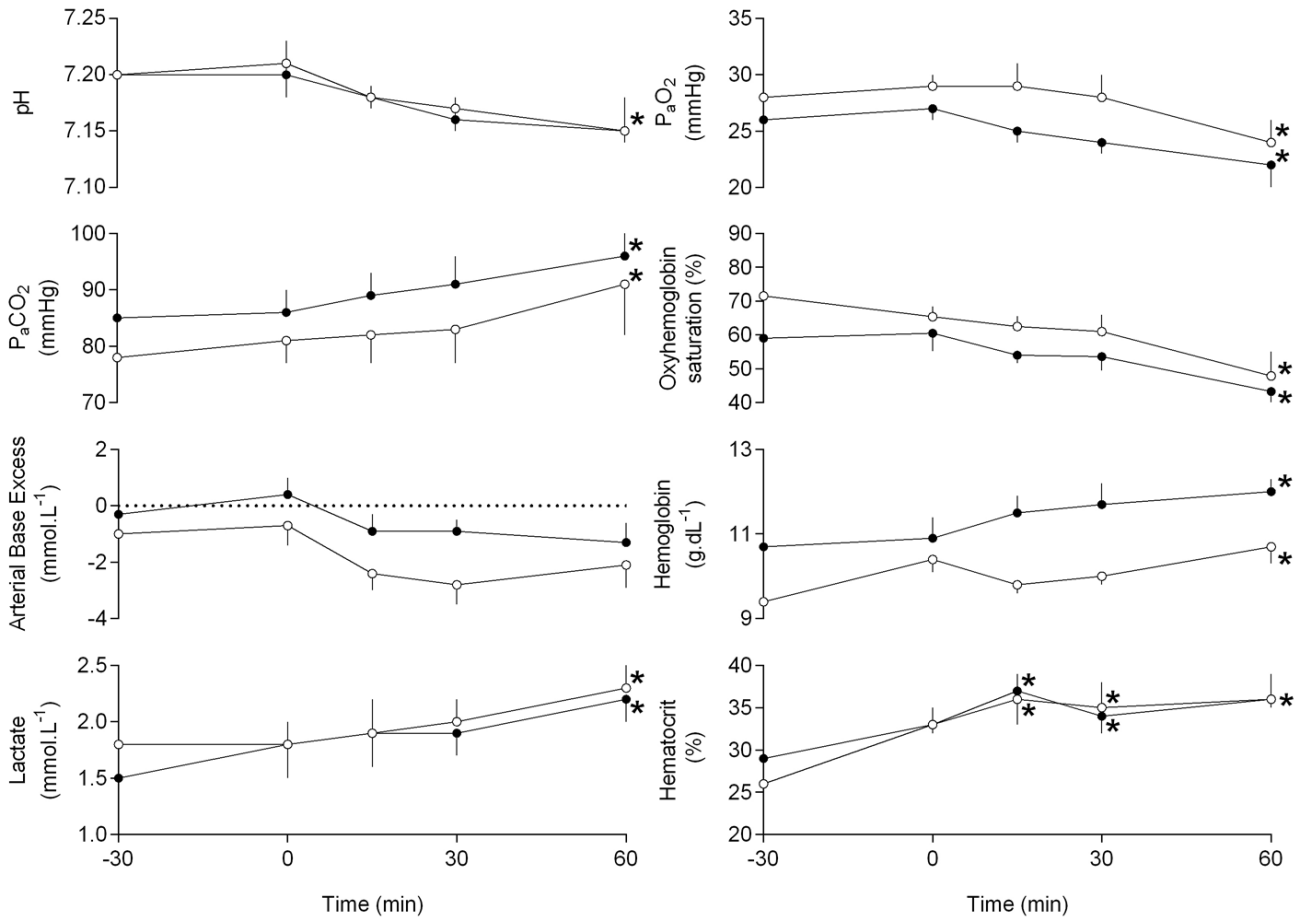
Fetal arterial acid–base and metabolic status. Values represent mean ± SEM of n = 9 for fetal carotid (open circles) and femoral (closed circles) arterial blood samples at the start (“− 30”) and end (“0”) of the baseline period; the midpoint (“15”) and end (“30”) of the period of uterine handling and the end of the recovery period (“60”). Significant differences are: *P < 0.05, for each timepoint when compared to timepoint “− 30”, assessed using a repeated-measures (RM) one-way ANOVA with post hoc Bonferroni test. The *P* value quoted in the final column is the overall significance of the RM ANOVA with Glasshouse–Geisser correction for the effect of time.

During the baseline period, values for fetal arterial blood gas and acid base status were stable and appropriate for this breed of sheep under prolonged general isofluorane anesthesia (Fig. 3). During uterine handling all values for fetal arterial blood gases and acid base status remained unaltered from baseline, with the exception of a significant increase in fetal hematocrit in both the carotid and femoral circulations (Fig. 3). During recovery, fetal pH, P_a_O_2_ and oxyhemoglobin saturation reached significantly reduced values compared to baseline. Conversely, during recovery, fetal P_a_CO_2_, blood lactate, hemoglobin concentration and hematocrit reached significantly elevated values compared to baseline (Fig. 3).

During the baseline period, values for calculated oxygen delivery in the carotid and femoral circulation were stable and appropriate for this breed of sheep under prolonged general isofluorane anesthesia (Table 2). During uterine handling, while carotid oxygen delivery was maintained, there was a significant fall in femoral oxygen delivery 15 min after the onset of uterine manipulation (Table 2). However, by the end of the uterine handling period, femoral oxygen delivery had partially recovered towards baseline values. During recovery, values for calculated oxygen delivery in the carotid and femoral circulation were unaltered from baseline (Table 2).

**Table 2:** Fetal oxygen delivery to pre and post ductal circulations.

Variable	Baseline		Uterine handling		Recovery	*P* value
	(− 30 min)	(0 min)	(15 min)	(30 min)	(60 min)	
Carotid oxygen delivery (mmol min^−1^)	337 ± 57	340 ± 35	300 ± 27	309 ± 36	251 ± 27	0.21
Femoral oxygen delivery (mmol min^−1^)	102 ± 14	106 ± 15	73 ± 11*	86 ± 13	77 ± 17	0.01

Values represent mean ± SEM for n = 9 of fetal carotid and femoral arterial oxygen delivery calculated at the start (“− 30”) and end (“0”) of the baseline period; the midpoint (“15”) and end (“30”) of the period of uterine handling and the end of the recovery period (“60”). Significant differences are: *P < 0.05, for each timepoint when compared to timepoint “− 30”, assessed using a repeated-measures (RM) one-way ANOVA with post hoc Bonferroni test. The *P* value quoted in the final column is the overall significance of the RM ANOVA with Glasshouse–Geisser correction for the effect of time.

## Discussion

The data show that direct intra-operative handling of the uterus in pregnant sheep in late gestation results in an acute fall in uterine artery blood flow, unrelated to the duration of anesthesia. This change in uterine arterial blood flow was associated with fetal peripheral vasoconstriction, which was acute in onset but persisted beyond the duration of the uterine insult. Notably, the fetal peripheral vasoconstriction during uterine manipulation occurred without evidence of fetal hypoxia and fetal heart rate and fetal carotid blood flow remained unaltered from baseline. Consequently, the fetal brain-sparing index, the ratio of parallel changes in blood flow between the carotid and femoral circulation^5, 10^, increased during uterine manipulation by virtue of the fall in femoral blood flow. The reduction in femoral blood flow led to a fall in oxygen delivery to the fetal peripheral circulation after the onset of uterine manipulation. By the end of the uterine handling period, the fetal peripheral vasoconstriction was sufficient to worsen the accumulation of lactate and acidosis in fetal blood.

In the present study, values for basal uterine blood flow and basal maternal arterial blood pressure during general anesthesia were lower than values measured in conscious pregnant sheep^8^. Intraoperative reductions in uterine arterial blood flow and maternal arterial blood pressure in pregnant subjects under general anesthesia are common and this has been previously reported in humans and sheep^11–18^. In the present study, the acute fall in uterine artery blood flow associated with uterine handling was independent of the effects of anesthesia on basal maternal arterial blood pressure or uterine blood flow or acute changes in maternal arterial blood gas status. The sudden increase in uterine artery vascular resistance may therefore be indicative of vascular spasm in the uterus. There is currently no published data, which relates uterine blood flow to uterine handling in human pregnancies. Of note in the present study, these changes in uterine blood flow occurred in the absence of deliberate sustained compression or rotation of the uterus, with a degree of contact that approximated the routine handling or packing of the uterus, which could occur during abdominal surgery in pregnant women. The most common operations in the cohort study of 6.5 million women which showed an increase in adverse outcomes following abdominal compared to non-abdominal surgery^1^ were appendicectomy followed by cholecystectomy, during which 20–40 min of intra-abdominal surgery is typical, roughly approximating to our 30 min of uterine manipulation.

The hemodynamic and metabolic responses in the fetus associated with uterine manipulation are interesting, as they resemble to some extent, the fetal cardiovascular and metabolic defense responses to acute hypoxia^4, 5, 10^. The physiology underlying these fetal compensatory responses to acute hypoxia is well established and it has been delineated from experiments in chronically instrumented fetal sheep in late gestation^4, 10^. In response to acute hypoxia, there is a redistribution of the fetal cardiac output away from peripheral circulations, such as the femoral vascular bed, towards more essential vascular beds, such as those perfusing the fetal brain. The fall in fetal femoral blood flow involves neural mechanisms, being triggered by a carotid chemoreflex and mediated by activation of the sympathetic nervous system, which promotes peripheral vasoconstriction^4, 10^.

Fetal lactic acidemia during acute hypoxia results from anaerobic metabolism of glucose in hypoxic fetal tissues, particularly in the hind limb muscle where blood flow and oxygen delivery are markedly reduced^19, 20^. Decreased oxygen delivery to the hind limbs increases lactate output, acidifying the fetal blood, which acts as a compensatory response when not too severe, facilitating the unloading of oxygen from hemoglobin to the fetal tissues^19, 20^. At the start of the baseline and uterine manipulation periods in our experimental protocol, values for fetal arterial lactate are stable and within normal limits, measuring 1.8 mmol l^−1^ in both the carotid and femoral arteries. However, once the onset of uterine manipulation promotes peripheral vasoconstriction and redistribution of blood flow, the fetal arterial lactate begins to rise reaching statistical significance 60 min later, with values of 2.3 mmol l^−1^ and 2.2 mmol l^−1^ in the carotid and femoral arteries, respectively. Raised umbilical arterial lactate, of which femoral arterial lactate is representative in our model, is a marker of both birth asphyxia and a predictor of adverse neonatal outcomes in human pregnancy. A recent systematic review of over 38,000 pregnancies reported that umbilical artery lactate measurements of > 3.2–3.9 mmol l^−1^ can predict hypoxic ischemic encephalopathy (HIE) and poor neonatal outcome with sensitivity and specificity equivalent to umbilical artery pH and base excess (pH < 7.0–7.15 and BE < -8 are typically used)^21^. Therefore, in our animal model, the duration or magnitude of uterine manipulation appears to be insufficient to produce arterial lactate changes predictive of birth asphyxia or poor neonatal outcome, but the trend suggests that more prolonged or aggressive uterine manipulation could be of concern.

While fetal brain sparing circulatory responses have been reported during compression of the uterine artery in ovine pregnancy leading to much greater falls in uterine artery blood flow and in fetal oxygenation^22, 23^, in the present study, uterine manipulation triggered femoral vasoconstriction without a fall in fetal oxygenation to hypoxic levels or those sufficient to trigger a fetal carotid chemoreflex^4, 10^. Further data in the present study support that the fetal femoral vasoconstriction in response to uterine manipulation is mediated via mechanism independent of fetal hypoxia and/or activation of the fetal carotid chemoreflex, since fetal heart rate was maintained throughout. In response to acute hypoxia, the fetal carotid chemoreflex also triggers fetal bradycardia, which is mediated via vagal efferent pathways^4, 24, 25^. The fetal bradycardic and femoral vasoconstrictor responses to acute hypoxia are part of the same carotid chemoreflex, since both can be abolished by bilateral carotid chemodenervation^4, 10^. Hence, dissociation of the fetal heart rate and femoral blood flow responses to uterine manipulation is further strong evidence that the femoral vasoconstriction in the present study is mediated via mechanisms other than those triggered by fetal acute hypoxic stress. It is also important to highlight that the magnitude of the increase in femoral vascular resistance in response to uterine manipulation in the present study (*ca.* 70% above baseline) is much smaller than in response to moderate fetal hypoxia. Data derived from the same breed of sheep as used in the present study at corresponding gestational ages show that the increase in femoral vascular resistance in response to acute hypoxia of the type that will reduce the fetal arterial P_a_O_2_ by half to values of 10–12 mmHg in the descending aorta is *ca.* 300–400% above baseline^4, 24, 25^. It is also interesting that uterine handling in the present study was associated with a significant increase in fetal, and to a lesser extent in maternal, hematocrit. In sheep, during the fetal and adult periods, the spleen can act as a reservoir of red blood cells^26^. Sympathetic stimulation of splenic contraction and other venous reservoirs will thus decrease venous capacitance and shift blood fast into the systemic circulation. This will promote an acute increase in red blood cell count and, thereby, hematocrit, as seen in the present study. Therefore, the changes in red blood cell concentration further support the idea that uterine handling in the present study is associated with increased fetal sympathetic outflow as a result of a mechanical stimulus, triggering femoral vascular and splenic constriction akin to the reflex triggering vagal-mediated fetal decelerations attributed to a mechanoreceptor response to compression of the fetal head^27^.

Additional data in the present study show accumulation of carbon dioxide in the maternal arterial circulation during general anesthesia, which has important implications for fetal blood gas exchange and acid–base status of further relevance to human obstetric surgery. It is well established that well oxygenated blood has a reduced affinity for carbon dioxide, displacing CO_2_ from hemoglobin and promoting its clearance; the so-called Haldane effect^28^. During pregnancy, as fetal blood flows through the placenta, oxygen will diffuse from the maternal into the fetal circulation, displacing CO_2_ from fetal hemoglobin. This enhances the feto-maternal P_a_CO_2_ gradient and thereby fetal CO_2_ clearance by the placenta. Elegant mathematical models by Longo and his group have calculated that during pregnancy, the Haldane effect accounts for ca. 46% of the CO_2_ exchanged in the placenta^29^. Clearance of fetal CO_2_ across the placenta is also limited by uterine blood flow^29^. In the present study, ventilation of the mother with a higher fraction of inspired oxygen during general anesthesia, will have a greater oxygenation and thereby Haldane effect in the maternal compared with the fetal blood. Therefore, during general anesthesia, a greater displacement of CO_2_ in the maternal blood coupled with impaired basal uterine blood flow and less effective maternal CO_2_ pulmonary clearance resulting from the recumbent position, will all contribute to a diminished feto-maternal transplacental transfer of CO_2_, thereby promoting a progressive increase in fetal PaCO_2_. Fetal hypercarbia coupled with increased fetal lactate output by the hind limbs, resulting from the reduced femoral oxygen delivery during uterine manipulation^19, 20^ has an important effect on fetal pH levels, which worsen from an already compromised baseline by the end of general anesthesia. Prolonged general anesthesia involving uterine manipulation in human obstetric practice will therefore support a similar effect on fetal blood gas status.

A strength of this study is that it demonstrates the potential consequences of both uterine handling and prolonged general anesthesia in a model of abdominal surgery during pregnancy. The pregnant sheep model is unique in this sense, as it permits invasive parallel collection of fetal cardiovascular and metabolic data directly in a robust preparation, which cannot be achieved by non-invasive methods in the human fetus. No other animal model has been developed to the same extent. However, there are several limitations of this study which also need to be considered. First, the mechanisms mediating the fall in uterine artery blood flow and the fetal femoral vasoconstriction remain to be determined. This demonstrates an area in which the knowledge of the field about mechanical effects on utero-placental function and their relationship to fetal physiology is lacking and suggests a potential rich avenue for further research. Second, the ovine uterus is much more tolerant to fetal surgery than the human or non-human primate. The consequences of uterine manipulation are therefore likely to be a greater risk factor for adverse fetal outcomes in the primate rather than the ovine fetus. Third, the experimental protocol was performed in ewes directly following the insertion of maternal and fetal catheters and flow probes. These are procedures which needed both uterine and fetal surgical manipulation prior to the uterine manipulation described in the experimental protocol, thereby prolonging the duration of general anesthesia delivered to the ewe overall. While values for uterine and fetal carotid and femoral blood flow were all within normal limits at the start of the baseline period in the present study, there was a trend to fetal hypercapnia and respiratory acidosis which would not be expected in a fetus at the start of maternal surgery. Therefore, it cannot be assumed that the fetal neuroendocrine compensatory response to uterine manipulation under these circumstances might be the same compared to the one triggered by an undisturbed fetus without prolonged exposure to preceding anesthesia.

In conclusion, data in the present study show that in an ovine model of pregnancy in late gestation, uterine handling under general anesthesia has the potential to produce significant reductions in uterine blood flow, triggering fetal peripheral vasoconstrictor responses, which may worsen fetal acidosis, even in the absence of fetal hypoxia. This may provide a potential explanation linking reported adverse obstetric outcomes in abdominal compared to non-abdominal surgery during pregnancy. Such information is important for fetal intervention patients, as operative procedures during late gestation under general maternal anesthesia become more prevalent.

## Supplementary information

Supplementary file 1 (DOCX 103 kb)
