# Supplementary material for: Maternal and fetal cardiovascular and metabolic effects of intra-operative uterine handling under general anesthesia during pregnancy in sheep
Source: Sci Rep. 2020 Jul 2;10:10867. doi: 10.1038/s41598-020-67714-y (PMC7331497; doi:10.1038/s41598-020-67714-y)
Supplement: Supplementary file 1 — (DOCX 103 kb) [file 41598_2020_67714_MOESM1_ESM.docx]

## Maternal and fetal cardiovascular and metabolic effects of intra-operative uterine handling under general anesthesia during pregnancy in sheep

**Authors:** Caroline J. Shaw, PhD ^1,2^, Kimberley J. Botting, PhD ^1,3^, Youguo Niu, PhD^1,3^, Christoph C. Less, MD ^2,4^ & Dino A. Giussani, PhD ^1,3^

Supplementary material


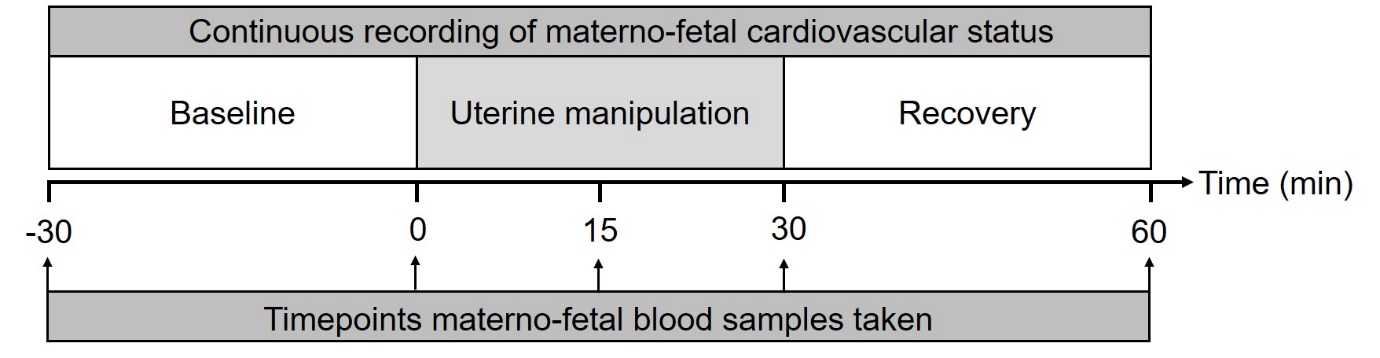


Figure s1: Timeline of experimental procedures and assessment of materno-fetal wellbeing

The diagram shows the experimental timeline, divided into periods of baseline, uterine manipulation and recovery. The frequency and timing of assessments of cardiovascular and acid-base status of mother and fetus are also indicated.
